# Organic Amendments for Mitigation of Salinity Stress in Plants: A Review

**DOI:** 10.3390/life12101632

**Published:** 2022-10-18

**Authors:** Md. Najmol Hoque, Shahin Imran, Afsana Hannan, Newton Chandra Paul, Md. Asif Mahamud, Jotirmoy Chakrobortty, Prosenjit Sarker, Israt Jahan Irin, Marian Brestic, Mohammad Saidur Rhaman

**Affiliations:** 1 Department of Biochemistry and Molecular Biology, Khulna Agricultural University, Khulna 9100, Bangladesh; 2 Department of Agronomy, Khulna Agricultural University, Khulna 9100, Bangladesh; 3 Department of Genetics and Plant Breeding, Bangladesh Agricultural University, Mymensingh 2202, Bangladesh; 4 Department of Agricultural Chemistry, Khulna Agricultural University, Khulna 9100, Bangladesh; 5 Department of Soil Science, Khulna Agricultural University, Khulna 9100, Bangladesh; 6 Department of Crop Botany, Khulna Agricultural University, Khulna 9100, Bangladesh; 7 Department of Botany and Plant Physiology, Czech University of Life Sciences, Kamycka 129, 16500 Prague, Czech Republic; 8 Institute of Plant and Environmental Studies, Slovak University of Agriculture, A. Hlinku 2, 94976 Nitra, Slovakia; 9 Department of Seed Science and Technology, Bangladesh Agricultural University, Mymensingh 2202, Bangladesh

**Keywords:** bio-fertilizer, ionic homeostasis, organic amendments, salinity, vermicompost

## Abstract

Natural and/or human-caused salinization of soils has become a growing problem in the world, and salinization endangers agro-ecosystems by causing salt stress in most cultivated plants, which has a direct effect on food quality and quantity. Several techniques, as well as numerous strategies, have been developed in recent years to help plants cope with the negative consequences of salt stress and mitigate the impacts of salt stress on agricultural plants. Some of them are not environmentally friendly. In this regard, it is crucial to develop long-term solutions that boost saline soil productivity while also protecting the ecosystem. Organic amendments, such as vermicompost (VC), vermiwash (VW), biochar (BC), bio-fertilizer (BF), and plant growth promoting rhizobacteria (PGPR) are gaining attention in research. The organic amendment reduces salt stress and improves crops growth, development and yield. The literature shows that organic amendment enhances salinity tolerance and improves the growth and yield of plants by modifying ionic homeostasis, photosynthetic apparatus, antioxidant machineries, and reducing oxidative damages. However, the positive regulatory role of organic amendments in plants and their stress mitigation mechanisms is not reviewed adequately. Therefore, the present review discusses the recent reports of organic amendments in plants under salt stress and how stress is mitigated by organic amendments. The current assessment also analyzes the limitations of applying organic amendments and their future potential.

## 1. Introduction

Soil salinity is a key abiotic stress that interferes with crop growth, development, and yield through altering morphological, physio-biochemical, and molecular processes [1,2,3,4,5,6]. Every year, 1–2% of cultivable land is reduced due to soil salinity and worldwide, about 800 million hectares (23%) of total arable lands are affected by soil salinity [7,8]. It is predicted that salinity will affect 50% of the world’s arable land by 2050 [9]. It has been reported that the rise in groundwater levels with high salt content, inefficient drainage and irrigation systems, and the overuse of fertilizers are responsible for soil salinity [10]. Plants use a number of methods to counteract salt stress in order to survive in an ever changing environment. Metabolic adjustments, increasing Na^+^ efflux or Na^+^ compartmentalization to vacuoles, scavenging of free radicals, the safeguarding of cellular machinery, ionic homeostasis maintenance, certain proteins expression and the up-regulation of their genes and so on are the plant adaptation mechanisms to salinity stress [11,12,13,14]. Additionally, it is widely recognized that using microRNAs (miRNAs) is a significant tactic that can affect post-transcriptional gene regulation under a variety of environmental conditions, including salt. Salt stress interaction is strongly controlled by post-translational gene regulations because various gene transcripts are differentially regulated by miRNAs during salt stress [15]. Furthermore, microRNAs serve important roles in embryogenesis, morphogenesis, life cycle stage transformation, flower formation, increases fruit ripening, boosts anthocyanin production, vegetative and reproductive stage transitions, tillering and branching, and enhances salinity stress tolerance in plants [15,16,17,18].

To reduce excess soil salinity, plant scientists are employing techniques such as sub-soiling, mixing sand, seed bed preparation, and salt scraping, as well as modern agronomic practices, hydrophilic polymer, gypsum, sulfur acids, green manuring, humic substance, farm yard manures, irrigation system, and salt-tolerant crops [19,20,21,22]. Recently, different organic amendments such as the application of vermi-compost (VC), vermi-wash (VW), biochar (BC), plant growth promoting rhizobacteria (PGPR), and bio-fertilizers (BF) are being used widely to ameliorate the negative consequences of soil salinity [5,6,23,24,25,26]. For instance, VC enhances morphological traits, chlorophyll content, antioxidant enzyme activities, and improves salinity tolerance of maize plants [27]. Several studies showed that BF and BC enhance plant growth progressions under salinity stress by improving antioxidant enzyme activities, and reduces oxidative damage in different plants [5,28,29]. In addition, the inoculation of PGPR under salt stress accelerates microbial population and gene expression in the rhizosphere, boosts biomass production and enhances the salt tolerance of different plants [6,30,31]. The organic amendments mitigate salt stress via a wide range of mechanisms, including the regulation of ionic homeostasis, antioxidant enzyme activities, and the reduction of oxidative damage. Several studies described that PGPR and BC relieved the negative effects of salinity by increasing the photosynthetic rate, antioxidant enzyme functions, secondary metabolites accumulation, and decreasing ROS in plants [6,32,33,34]. Organic amendments such as VC and VW include a variety of plant growth-regulating components such as micro and macro elements, vitamins, enzymes, and hormones that have been shown to reduce the harmful effects of salts on plants [25]. Furthermore, several studies have stated that VC and VW have been shown to reduce soil salinity through the enhancement of antioxidant enzymes and to lessen electrolyte leakage and oxidative stress [35,36]. Similar to other organic supplements, BF has been shown to attain a better environment through fixing atmospheric nitrogen, phosphate and potassium solubilization or mineralization, releasing o plant growth regulating materials, producing antibiotics, and degrading organic matter in the soil, all of which contribute to increased plant salinity stress tolerance [37,38,39].

The world’s population grows significantly every day. To feed the increasing millions, researchers are attempting to develop modern, effective agronomic and eco-friendly organic ways to reduce salinity stress and boost crop yields. Furthermore, investigating environmentally safe and sustainable methods to lessen the negative consequences of salinity is necessary due to the ongoing environmental degradation. The literature suggests that organic approaches can alter biochemical and molecular systems to enable plants to withstand salinity stress, and these strategies are proving to be quite effective. As a result, this report highlighted the recent findings about organic amendments like VC, VW, BC, BF, and PGPR used for salinity stress mitigation. Correspondingly, keeping in view the role of organic amendments during saline conditions, this review explores the potentiality of the modern and widely used organic amendments for the alleviation of salt stress and their regulatory mechanisms. Despite the fact that other reviews of organic amendments for reducing salt stress have been published independently, this study offers a thorough analysis of all commonly used organic amendments for reducing salt stress in a single frame.

## 2. Organic Amendments for Salinity Stress Mitigation

### 2.1. Vermicompost and Vermiwash

The VC is an organic fertilizer that is prepared through the conversion of organic wastes by worms [40], and is rich in different enzymes including humic and fulvic acids [41]. It contains a number of plant growth regulating substances (micro and macro elements, vitamins, enzymes, and hormones) and has anti-stress effects [42,43]. Earthworms in VC have beneficial effects on soil qualities such as physical, chemical, and biological properties [44], as well as increasing plant development and production by making nutrients available to the plant [45].

**Figure 1:**
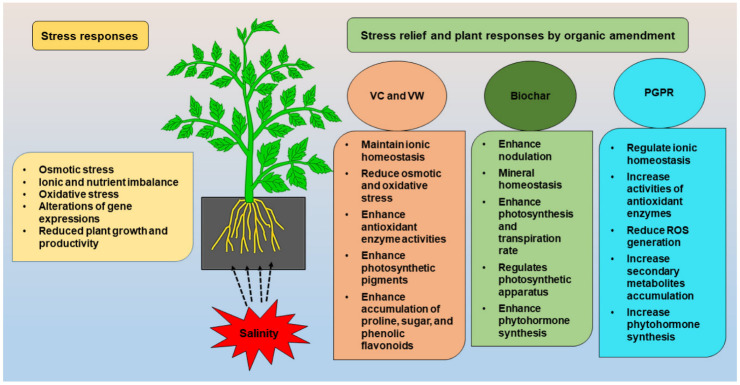
Stress relief and possible plant responses by VC, VW, BC, and PGPR.

The VW and vermicompost leachate (VCL) are two important derivatives prepared from vermicompost. The VW is a clear, translucent, pale-yellow fluid obtained by passing water through a column of the vermi-worms’ excreta, which contains mucus secretions as well as micronutrients from decomposed organic sources [46]. Khan et al. [47] reported that VW has been utilized as an organic fertilizer for plants, and is a rich source of amino acids, vitamins, N, P, Mg, Zn, Fe, Cu, auxins and cytokinins. The VCL is a liquid that is collected when water drains over decomposing solids [48]. This liquid may drain out from a traditional compost bin or a worm bin. Leachate is used as soil drench after dilution. VCL appears to be an effective and environmental friendly VC derivative for lowering salt’s harmful impact on bean seedlings [49]. It was demonstrated that vermicompost promoted seed germination, root and shoot growth, proline accumulation, and oxidative stress management (Figure 1, [50,51,52]). In addition, VCL alleviates salt stress by enhancing photosynthetic efficiency, promoting antioxidant enzyme activity, and reducing electrolyte leakage [42]. Among the various organic amendments practices, VC and VW are low-cost techniques to reduce the detrimental impact of salts on plants (Table 1, [53]). The VC has been shown to reduce salt toxicity and enhance the emergence rate and seedling growth of different plants [54,55]. The VC enhances soil organic matter in salt soils by decreasing electrical conductivity (EC), bulk density and increasing field capacity, saturated hydraulic conductivity, and cation exchange capacity [56].

A number of studies show that VC enhanced salinity tolerance and improved the morphological characteristics such as stem and root length, fresh and dry weight of root-shoot, vigor index, leaf area, and dry weight per plant [54,57,58,59]. It has been reported that VC boosted Na^+^ exclusion and K^+^ accumulation, alleviated stomatal restriction, raised leaf pigment concentrations, improved root activity, decreased oxidative damage in Fountain Grass, and improved salt tolerance [60]. In addition, VC application in maize and tomato plants under salt stress improved Chl a, Chl b, Total Chl, carotenoids, CAT, POD, and SOD and lowered H_2_O_2_ and MDA [27,61]. The addition of VC and VW to potato improved growth metrics, plant height, stem diameter, and tuber features, reducing the impact of salt stress [35]. Liu et al. [62] found that in coastal saline soil, VC application in maize increased nutrient availability and soil macro-aggregates by up to 91.02 percent. The soil amendment of VC increased exchangeable K^+^, Ca^2+^, and Mg^2+^, plant height, total dry matter content, and decreased exchangeable Na^+^ in the saline soil [63,64,65]. In addition, VC application reduced salt-induced injuries of plants grown in saline soil by increasing relative water content, stomatal conductance, chlorophyll-a, superoxide dismutase (SOD), ascorbate peroxidase (APX), and catalase (CAT) activities and decreased electrolyte leakage and malondialdehyde (MDA) levels [55,66,67,68].

**Table 1:** Application of vermi-compost (VC) and its derivatives for minimizing soil salinity.

Plant Species	Stress Level	Treatment and Application Methods	Effects of Amendments	References
Tomato (*Solanum lycopersicum* L.)	NaCl @ 150 mM	VC @ 6 mL/L	Improved foliar growth, increased water content of the leaves, reduced osmotic potential at the root level and Na content of the leaves; promoted the accumulation of proline and total sugars.	[69]
	NaCl @ 125 mM	VCL @ 18 mLL^−1^	Improved plant growth and lowered Na^+^ deposition in salt-stressed plants; delayed young leaf senescence.	[70]
	NaCl @ 0, 50 and 150 mM	VC @ 10, and 20%	Increased shoot length, stem diameter, leaves number, root length, shoot and root fresh, dry weight, Chl a, Chl b and carotenoid; increased Cat; decreased MDA; improved salinity tolerance.	[61]
Potato (*Solanum tuberosum* L.)	NaCl @ 15, 20, and 25 mM	VC @ 300, 580, and 860 g plant^−1^; VW @ 5-, 10-, and 15-mL plant^−1^	The addition of VC and VW increased the height of the plant and the diameter of the stem. VC reduced salinity effects on the plant.	[35]
	2.85 dSm^−1^	Exogenous VC, proline and glycine betaine	Increased growth, yield, bio-constituents and antioxidant enzymatic activity. Improved salt tolerance of potatoes	[71]
Maize (*Zea mays* L.)	NaCl @ 0, 50, 100, 150 and 200 mM	VC with bacteria having ACC deaminase activity	Improved seed germination and the growth of seedlings; increased proline, chlorophyll content and alleviated the salt stress.	[72]
	Coastal saline soil	VC with humic acid fertilizer	Increased soil macro-aggregates, improved soil nutrient availability and maize nutrient uptake.	[62]
	NaCl @ 6, and 12 dS m^−1^	VC @ 5, and 10%	Increased root, shoot fresh and dry weight; increased Chl a, Chl b, total Chl, carotenoids; increased CAT, SOD, POD activities; decreased H_2_O_2_, MDA content; increased salinity tolerance.	[27]
Moldavian dragonhead (*Dracocephalum moldavica* L.)	NaCl @ 0,50, 100 and 150 mM	VC @ 0, 5, 10 and 15% (*v*/*v*)	Increased plant biomass, chlorophyll content and proline accumulation. Reduced the effects of high sodium chloride concentrations.	[59]
Lemon verbena (*Lippia citriodora*)	NaCl @70 mM	VC @ 0%, 10% and 30% of pot volume	Alleviated salt stress by improving the growth and phenolic compounds of the plants.	[73]
Basil (*Ocimum basilicum* L.)	NaCl @ 0, 50 and 100 mM	Humates VC @ 0 and 1/60 *v*/*v*	Enhanced shoots and roots length, fresh and dry biomass of root, stem, leaf and leaf area. Reduced salinity.	[74]
Smoke tree (*Cotinus coggygria* Scop.)	NaCl @ 1, 4 and 7 dS.m^−1^	VC @ 80% *v/v* soil + 20% *v*/*v*	Increased fresh and dry weight of shoots, increased leaf area; Reduced sodium and chloride of leaf and increased potassium. Increased salt tolerance of plant.	[58]
Fenugreek (*Trigonellafoenum-graecum* L.)	NaCl @ 0, 100 and 200 mM	VC @ 0, 5 and 10 weight%	Increased number of seed per pod, number of pods, number of sub branch and plant height. Reduced salinity effects.	[57]
Wheat (*Triticum durum* Desf. cv. Yelken)	High salt stress	VC and fish flour (1:1)	Enhanced growth, seed vigor and total phenolic-flavonoids, chlorophyl-carotenoids contents, and increased phenylalanine ammonialyase (PAL), peroxidase (POD) activities. VC decreased salinity effects.	[75]
	Coastal salinity	Soil amendment of VC	Increased soil macro-aggregates; Improved shoot biomass, grain yield, soil physical, chemical and biological properties. Ameliorated salt-induced stress.	[76]
	Saline soil	VC @ 10.0-ton ha^−1^; Biochar @ 10-ton ha^−1^	Improved relative water content, total chlorophyll, stomatal conductance, leaf K^+^ concentration; Reduced oxidative stress, leaf Na^+^ concentration, and proline content; improved yield related traits, productivity, soil water level and chemical properties. Eliminated the detrimental effects of soil salinity.	[68]
Rice (*Oryza sativa*)	Soil salinity	VC and rice husk ash @ 1000 kg per Rai for both	Increased exchangeable K^+^, Ca^2+^ and Mg^2+^ in soil; reduced electrical conductivity and risen tillers per clump; improved the physiological and biochemical responses. Increased the rice growth in salt affected area.	[44]
Lettuce (*Lactuca sativa*)	NaCl @ 0, 4 and 8 dS m^−1^	VC @ 0, 2.5 and 5% (*w*/*w*)	Enhanced soil organic matter, available P, total N, available K and the cation exchange capacity of the soils; Increased field capacity, available water capacity, saturated hydraulic conductivity, total porosity, and aggregate stability; Decreased EC values and the bulk density of the soils.	[56]
	NaCl @ 8.32 dS/m	VC 50% and pulverized eggshell 12.5%	Decreased soil salinity for about 77%; fasten the seed germination and seedling growth.	[77]
	NaCl @ 4, 8 dSm^−1^	VC 5% (*w*/*w*)	Increased P, K, Mg, Fe, Mn and Zn concentrations; decreased Na contents. Reduced toxic effects of salinity on the plant.	[65]
	NaCl @ 4 and 8 dSm^–1^	VC @ 0, 2.5 and 5% (*w*/*w*)	Increased relative water content, stomatal conductance, chlorophyll *a* content; decreased electrolyte leakage, malondialdehyde (MDA) contents; increased superoxide dismutase (SOD) and catalase (CAT) activities. Alleviated the salt stress.	[67]
Pot marigold (*Calendula officinalis* L.)	NaCl @ 0, 50, 100, 150 and 200 mM	VC @ 0%, 5%, 10%, 15% and 20%	Increased the activity of the antioxidant system; increased proline and chlorophyll content. Reduced salinity impacts and boost-up yield.	[78]
Noni (*Morinda citrifolia* L.)	Salinity stress @ 0.5, 1.5, 3.0 and 4.5 dS m^−1^	Substrates with humus; 33.33 and 66.66% of humus	Decreased the intensification of electrical conductivity of irrigation water; mitigated the negative effects of salts on plants.	[79]
Bean (*Phaseolus vulgaris* L.)	NaCl @ 20, 40, 60 and 80 mmol l^−1^	VC: Sand = 0:100; 10:90; 25:75; 50:50 and 75:25	Increased photosynthetic rate and potassium (K^+^) and calcium (Ca^2+^) concentration in leaf and root; improved the growth of bean plants. Alleviated negative effects of salinity.	[49]
Pomegranate (*Punica granatum* L.)	NaCl @ 0, 30, and 60 mM	Vermicompost leachate (VCL) foliar spray	Leaf area, photosynthetic efficiency, and shoot and root fresh and dry weight significantly increased; improved the activity of antioxidant enzymes; reduced oxidative stress and electrolyte leakage. VCL alleviated the damage caused by salt stress	[42]
Tall fescue turfgrass (*Festuca arundinacea* cv Queen)	NaCl @ 0, 3, 6 and 12 dS/m	VC @ 0, 100, 200 and 300 g	Activities of CAT and APX were increased; leaf area, shoot length and dry shoot weight were highest. Reduced the effects of high concentrations of sodium chloride in saline soils.	[66]
Onion (*Allium cepa* L. cv. Metan)	NaCl @ 50 and 100 mM	Seed Priming with VC	Higher germination, seedling growth, CAT, SOD and APX activities were found in VC treated seeds. VC mitigated salinity effects	[55]
Bell pepper (*Capsicum annuum* L.)	NaCl @ 160 mM	Addition of 7 mL/L VCL	Increased sugar concentration in roots and proline content in leaves; increased leaf fresh weight. VCL enhanced the property of salt-stress resistance in bell peppers.	[52]
Medicago (*Medicago rigidula* L.)	NaCl @ 0, 50 and 100 mM	VC @ 0, 10, 20 and 30%.	Increased plant survival capacity, chlorophyll contents, shoot dry weight; maximize leaf area values.	[80]
Sunflower (*Helianthus annuus* L.)	EC: 0.5, 4.8 and 8.6 dS/m	VC @ 1 kg/pot	Increased plant growth, yield, nitrate and protein content; decreased sodium (Na^+^), chloride (Cl^−^), ammonium; Increased N-assimilation.	[64]
Borage (*Borago officinalis*)	NaCl @ 0, 4, 8 and 12 dSm^−1^	VC @ 0, 6, 12 and 18% (*w*/*w*) of soil	Increased chlorophyll b, carotenoids and MDA contents and reduced the negative effects of salinity.	[81]
Milk thistle (*Silybum marianum* L.)	NaCl @ 0, −2, −4, −6, and −8 bar	Superabsorbent polymers with VC coats	Increased seedlings emergence rate, plant vigor index, shoot dry weight, leaf area, specific leaf area, relative water content, and total chlorophyll.	[54]
Sugarcane commercial variety of ‘Bululawang (BL)’	NaCl @ 4.12 dS/m	VC @ 0, 10, 20 t/ha) and nitrogen fertilizer @ 50, 75 and 100 kg N/ha	Increased N, K uptakes and the growth of sugarcane and alleviated salinity effects.	[82]
Rapeseed (*Brassica napus* L.)	NaCl @ 100 mM	VCL (1:10, *v*/*v*)	VCL was shown to improve seed germination and management of oxidative stress.	[51]
Fountain Grass (*Pennisetum alopecuroides*)	NaCl @ 5.0 g per kg soil	VC	Enhanced Na^+^ exclusion and K^+^ accumulation, relieved stomatal limitation, increased leaf pigment contents, enhanced electron transport efficiency and net photosynthesis, improved root activity, and minimized the oxidative damage.	[60]

### 2.2. Biochar

BC is a carbon-rich organic substance with a porous structure, a wide surface area, and a high ion exchange ability that improves the physical qualities of agricultural soil [83,84]. A number of studies found that BC application improves the different physio-biochemical processes such as photosynthesis, hormonal and enzymatic activity in plants and decreases the harmful effects of salt stress on plants (Figure 1 and Table 2, [5,22,83,84,85]).

Morphological attributes such as seedling emergence, plant height, shoot biomass, root biomass, leaf area, dry matter, and yield of plants under salinity stress have been shown to be improved by BC incorporation [32,86,87]. Moreover, BC application boosted photosynthetic rate, stomatal conductance, and transcription rate under salinity stress conditions in wheat [84], sorghum [87], quinoa [83], and eggplant [32]. On the other hand, the availability and uptake of different nutrients such as N, P, K in maize [88] and P, K, Fe, Mn, Zn, and Cu in tomato [89] improved by the utilization of BC as amendment to saline soil.

Furthermore, in saline conditions, BC traps excess Na^+^ in soil, releasing mineral nutrients and decreasing osmotic stress [86]. Studies showed that the use of BC lowered the concentration of Na^+^ and decreased the Na^+^/K^+^ ratio in a variety of plants, assisting in the reduction of the negative effects of salt on plants [84,90]. Moreover, under salinity stress, BC application improves osmotic balance by increasing water holding capacity and CO_2_ assimilation, which ultimately results in a better photosynthetic rate, stomatal conductance, and transcription rate [32,83,86]. It has been reported that the leaf photosynthesis and net assimilation rate of the rice population was greatly aided by biochar’s potential positive effects on chlorophyll content, leaf N content, leaf area index, photosynthetic potential, stomatal conductance, and transpiration rates [91,92]. Additionally, the biochar treatment greatly enhanced the salt tolerance of cabbage seedlings and dramatically raised LRWC, Chl a, Chl b, and total Chl while reducing sucrose, proline, H_2_O_2_, and MDA [29]. In addition, BC application reduced the effects of salt by lowering the levels of phytohormones such ABA, ACC, and JA, as well as increasing the amount of IAA in beans [93]. Similarly, Nikpour-Rashidabad et al. [94] reported that BC improved the vascular cylinder, parenchyma, IAA/ABA and IAA/ACC ratios to ameliorate the effects of salinity. Furthermore, under salt stress, the treatment with BC enhanced nodulation, nitrogen content, rubisco activity, GDH, GS, GOGAT, and NR activities in various parts of the soybean plant [85]. Given the findings of the preceding investigations, BC appears to be a promising strategy for reducing salt stress and increasing plant growth and biomass in a variety of plants.

**Table 2:** Application of biochar (BC) for minimizing soil salinity.

Plant Species	Stress Level	Treatment and Application Methods	Effects of Amendments	References
Wheat (*Triticum aestivum* L.)	Saline water irrigation @ 10 dSm^−1^	BC @ 10, 20, 30 t/ha	Increased and relative water content, photosynthesis. Decreased Na^+^/K^+^, and leaf senescence.	[84]
	NaCl @ 3000 ppm	Soybean straw BC	Increased plant growth, grain yield and biomass production; increased leaf chlorophyll content, water use efficiency, PSII efficiency, and net photosynthesis rate; decreased electrolyte leakage, H_2_O_2_, MDA; increased CAT, APX, SOD, GR activities; improved salinity tolerance.	[95]
Quinoa (*Chenopodium quinoa* L.)	Saline water irrigation @ 400 mM	BC@ 5% (*w*/*w*)	Increased photosynthesis, stomatal conductance, WUE and K^+^ content. Decreased ABA and Na^+^ content.	[83]
Eggplant (*Solanum melongena* L.)	Saline water irrigation @ 2 and 4 dSm^−1^	Hardwood BC @ 5%, Softwood BC @ 5%	Increased biomass, photosynthesis and stomatal conductance. Decreased leaf temperature and electrolyte leakage in leaf tissue.	[32]
Maize (*Zea mays* L.)	Saline soil	Wheat straw BC @ 12 t/ha	Increased LAI, Chlorophyll content, K, P and N content. Reduced MDA, soluble sugar, ascorbic acid and proline content.	[88]
	Saline soil	BC @ 5% (*w*/*w*)	Increased photosynthesis and stomatal conductance, K^+^ content and K^+^/Na^+^. Decreased ABA and Na^+^ content.	[96]
Soybean (*Glycine max* L.)	NaCl @ 5 and 10 dSm^−1^	BC @ 50 and 100 g kg^−1^ soil	Improved nodulation, chlorophyll content, N content, rubisco activity, GDH, GS, GOGAT, and NR activities.	[85]
Bean (*Phaseolus vulgaris* L.)	NaCl @ 6 and 12 dSm^−1^	BC @ 10% and 20% *w*/*w*	Decreased Na^+^ concentration, PAO activity, polyamines, ABA, ACC and JA; enhanced IAA content.	[93]
Mungbean (*Vigna radiata* L.)	NaCl @ 5 and 10 dS m^−1^	BC @ 50 and 100 g kg^−1^	Increased and relative water content, IAA content, vascular cylinder, cortical parenchyma areas, IAA/ABA and IAA/ACC ratios; decreased ABA and ACC.	[94]
Sorghum (*Sorghum bicolor* L.)	NaCl @ 0.26, 5.8 and 12.6 dSm^−1^	Soil mixer @ 2.5%, 5% and 10% (*w*/*w*) of total mass	Increased photosynthesis, stomatal conductance, transpiration rate CAT, POD, and SOD activity.	[87]
	NaCl @ 0.8, 4.1, and 7.7 dS m^−1^	BC @ 0, 2.5, 5, and 10% (*w*/*w*)	Increased saddling emergence percentage, dry matter accumulation, and relative water content. Mitigated salinity stress.	[86]
Potato (*Solanum tuberosum* L.)	NaCl @ 25 and 50 mM	BC @ 5% *w*/*w* of total mass	Increased photosynthesis, stomatal conductance, leaf water potential, K^+^ content; decreased Na^+^, Na^+^/K^+^ ratio and ABA concentration.	[97]
Rice (*Oryza sativa*)	Saline soil	BC @ 0%, 1.5%, 3.0% and 4.5% *w*/*w*	Increased biomass, grain yield; decreased in leaf Na^+^ concentration and Na^+^/K^+^ ratio; increased in leaf K^+^ concentration; decreased ABA, MDA content; increased leaf photosynthesis rates (Pn), transpiration rates (Tr), stomatal conductance (Gs); improved salinity tolerance.	[91]
	NaCl @ 3 g per kg soil	BC application	Decreased the value of EC, soluble Na^+^ and Cl^−^ contents; increased CEC, SOM, HA, total nitrogen, and total phosphorus contents in the soil; increased soil microbial community.	[92]
Cabbage (*Brassica oleracea*)	NaCl @ 0 and 150 mM	BC @ 0%, 2.5%, and 5%	Increased stem diameter, leaf area, shoot fresh weight, root fresh weight, shoot dry weight, and root dry weight; decreased malondialdehyde (MDA), hydrogen peroxide (H_2_O_2_), proline, and sucrose content; reduced Cl and Na concentration, and reactive oxygen species (ROS) production; increased CAT and SOD activities.	[29]
Borage (*Borago officinalis*)	NaCl @ 1250, 2500, 5000, and 7500 mg per kg soil	BC @ 5%	Decreased leaf water potential (Yw), osmotic potential (Ys), water saturation deficit (WSD); increased relative water content (RWC), water content (WC), and water retention capacity (WTC); increased K^+^, and K^+^/Na^+^ ratio; decreased MDA, H_2_O_2_; increased POD, SOD activities; improved salinity tolerance.	[98]

### 2.3. Bio-Fertilizer

BFs are one kind of fertilizer that contains living cells from various microorganisms and can transform via biological mechanisms; nutrients are converted from the inaccessible to the accessible form [99,100]. Recently, many studies have described the potential of BF in salt tolerance enhancement (Table 3). The application of BF in wheat seedlings lessened the negative effects of salinity by increasing chlorophyll content and decreasing proline content, and improved plant growth and yield [28,37]. Under salt stress, amaranth enhanced plant height and biomass production [101]. It has also been reported that the application of BF to lavender enhanced its capacity to withstand salt stress by increasing morphological attributes and RWC, Chl a, Chl b, and total Chl content as well as its essential oil output [102]. Similarly, BF application on wheat (*Triticum aestivum* L.), okra (*Abelmoschus esculentus* L.), yellow passion fruit (*Passiflora edulis*), cowpea (*Vigna unguiculata* L.), corn (*Zea mays* L.), and olive plants (*Olea europaea* L.) enhanced growth and yield metrics, micro and macronutrient content, and relieved salinity-related detrimental effects [28,37,38,103,104,105,106]. Souza et al. [104] showed that in yellow passion fruit, BF application reduced the salt stress and enhanced the absolute growth rate, side branches, and yield. In addition, BF application to olive and papaya plants increased growth and plant biomass, improved osmotic adjustments between root and soil, increased microbial activity in the rhizosphere zone, and reduced the toxic effects of salts [106,107].

BF application increased antioxidant activity through the up-regulating of POX, SOD, and CAT, and reduced MDA and H_2_O_2_ production in lettuce (*Lactuca sativa* L.), safflower (*Carthamus tinctorius* L.) and cowpea (*Vigna unguiculata* L.) [105,108,109]. Al-Taey and Majid [108] found that the functions of POD, CAT, SOD, and MDA were increased as a result of the increased salinity stress in lettuce (*Lactuca sativa* L.). It has been reported that BF ameliorates the effects of salt stress via the production of phytohormones (IAA, CK, and ABA) and secondary metabolites (proline) in plants [109,110,111].

Overall, the discussion concluded that BFs increased plant growth and production while also inducing salt tolerance by enhancing antioxidant enzyme activities, secondary metabolite accumulation and phytohormone synthesis.

**Table 3:** Bio-fertilizer used for mitigating soil salinity.

Plant Species	Stress Level	Treatment and Application Methods	Effects of Amendments	References
Wheat (*Triticum aestivum* L.)	NaCl @ 0, 3000, 6000, 9000 ppm	Cerealien, Phosphorien and Cerealien + Phosphorien in addition mix-up with wheat grains.	Increased growth, dry matter accumulation, and yields. Decreased proline content. Improved salinity tolerance.	[37]
	NaCl @ 0, 2.76, 5.53, and 8.3 dSm^−1^	Four (04) biofertilizer treatments were applied: not at all biofertilizer; seed injection by *Azotobacter chroococcum* Beijerinck strain 5; *Pseudomonas putida* (Trevisan) Migula strain 186; joint inoculation of *Azotobacter* + *Pseudomonas*	Increased chlorophyll index, relative water content, and grain yield. Concentrated dry matter, stem reserve mobilizations to grain yield and decreased proline content.	[28]
Lettuce (*Lactuca sativa* L.)	Irrigated with saline water @ 1.2 dSm^−1^	Biofertilizer @ 5 kg/ha	Increased POD, CAT, MDA, SOD activities. Decreased disruption of endohormones, osmotic stress and mitigates salinity stress.	[108]
Geranium plant (*Pelargonium graveolens* L.)	Irrigated with saline water NaCl_1_: NaC1_2_ (1:1)	(Half dose of compost + Bio) & (full dose of peanut compost + Bio) added to the pot.	Increased oil percentage but N, P, K contents remained unchanged. Improved yield and mitigated salinity stress.	[112]
Okra (*Abelmoschus esculentus* L.)	Irrigated with saline water with 3 levels 0.47, 2, & 4 dSm^−1^	Biofertilizers + Ascorbic acid @ 100 & 200 mgL^−1^ was applied.	Increased chlorophyll content, growth and yield but deceased ascorbic acid and proline content in okra plants.	[38]
Barley (*Hordeum vulgare*) & Broad beans (*Vicia faba*)	Irrigated with saline water @ 0, −1, −3, −5 Mpa	Seeds were presoaked with biofertilizer (2 mL of nanomaterial + 10 mL cyanobacterial (algal culture) + 10 mL rhizobacterial strain + 10 mL MeSA) for one day and 12 h and then added to the saline soil.	Increased bioavailability of nutrients, production of growth hormones and bio-stimulants. Decreased Na^+^, Cl^−^, and proline concentrations ultimately reduced salinity.	[113]
Yellow passion fruit *(Passiflora edulis)*	Irrigated with saline water (EC 0.35 & 4 dSm^−1^)	Soil applied biofertilizer @ 0 and 50%	Increased absolute growth rate, period for pruning the side branches, and yield, and decreased the adverse effect of salinity.	[104]
Soybean (*Glycine max* L.)	Saline water @ 3.13, 6.25, 9.38 dSm^−1^	Seeds were inoculated with bio-fertilizers and applied on the field.	Increased ascorbic acid, total indoles, a- amylase activity and polyphenol oxidase, decreased total soluble phenols, total soluble sugars and free proline. Decreased the salinity effects.	[114]
Safflower (*Carthamus tinctorius* L.)	NaCl @ 250 mM	Coated seeds with biofertilizers & sugars were applied to the pot.	Increased antioxidant enzymes (SOD, CAT, POD, and APX), decreased proline and malondialdehyde (MDA). Improved salinity tolerance	[109]
Peanut (*Arachis hypogaea* L.)	Irrigated with saline water @ 0.5, 1.5, 2.5, 3.5, 4.5 and 5.5 dSm^−1^	Biofertilizer @ 15, 30 and 45	In the peanut, it promoted higher vegetative growth and improved photosynthesis rate. Decreased soil salinity and improved yield.	[115]
Cowpea (*Vigna unguiculata* L.)	NaCl @ 25, 50, 100, 200, and 300 mM	Biofertilizers mixed with sand @ 0.8 g/Kg	Increased growth parameters, total pigments, protein, proline contents and activities of SOD and CAT. Reduced H_2_O_2_ production and alleviated salinity stress.	[105]
Pitombeira seedlings (*Talisia esculenta*)	NaCl @ 0.8, 2, 4, 6, 8 dSm^−1^	Biofertilizer @ 10% of the total volume	Increased plant height, stem diameter, number of leaves, leaf area, total leaf area, Dickson quality index, dry mass of root and stem. Mitigated the harmful effects of salinity.	[116]
Cotton (*Gossypium hirsutum* L.)	NaCl @ 15 dSm^−1^	Seeds were coated with biofertilizers.	Increased shoot growth, root growth and yield. Decreased leaf gas exchange characteristics.	[117]
Corn (*Zea mays* L.)	Irrigated with saline water @ 0.47, 2.50, and 3.90 dSm^−1^	Biofertilizer “Halix” was applied as an inoculum to corn seeds before cultivation.	Increased the concentrations of macro and micronutrients, total chlorophyll, and ascorbic acid in maize plants, as well as mitigated the negative effects of salinity on corn.	[104]
Olive (*Olea europaea* L.)	Irrigated with saline water @ 2000, 3000 and 4000 ppm	Biofertilization treatments control, *Azotobacter* chroococcum, Mycorrhizae (*Glomus macrocarbium*) and mix of *Azotobacter chroococcum* + Mycorrhizae	Enhanced growth and plant biomass, improved microbial activity in the rhizosphere zone. Decreased intensity of salt toxic effects.	[106]
Papaya (*Carica papaya* L.)	Irrigated with saline water @ 0.5, 1, 2, 3 and 4 dSm^−1^	Biofertilizer applied @ 10% of the substrate volume.	Enhanced growth and plant biomass, provided greater osmotic adjustments between root and soil solution, increased absorption efficiency of water and essential nutrients stimulating plants to grow. Decreased intensity of salt toxic effects on growth.	[107]
Amaranth (*Amaranthus tricolor* L.)	NaCl @ 0, 2500, 5000, 7500, and 10,000 ppm	*Bacillus* sp., *Lactobacillus* sp., *Saccharomyces* sp., *Streptomyces* sp., *Azospirillum* sp., *Pseudomonas* sp., *Azotobacter* sp., *Rhizobium* sp.	Increased plant height, number of leaves, and stem metaxylem diameter.	[101]
Lavender (*Lavandula angustifolia*)	NaCl @ 0, 50, and 100 mM	Azotobacter, Azospirillum, and a combination of Azotobacter and Azospirillum	Increased plant height, stem length, root length, fresh weight, dry weight, relative water content, chlorophyll a, chlorophyll b, total chlorophyll, and essential oil yield; improved salinity tolerance.	[102]

### 2.4. PGPR

Plant-growth-promoting rhizobacteria (PGPR) are microorganisms that colonize plant roots and are used as chemical alternatives in agricultural fields for crop production and protection [6,118]. PGPR, which are resistant to salinity, help the plants to endure salty conditions. These plant-associated rhizobacteria can synthesize a variety of substances, including extracellular polymeric substance, 1-aminocyclopropane-1-carboxylate deaminase, phytohormones, antioxidants and volatile chemical compounds [6,30]. Gao et al. [119] reported that rhizosphere bacteria reduce salt stress while promoting plant development by supplying nitrogen, phosphate, potassium, auxin, cytokinin, and abscisic acid to plants. During several field tests, crops grown under saline soil conditions responded favorably to the utilization of PGPR in terms of growth and yield (Table 4). Kumawat et al. [120] in his study revealed that PGPR increased seed germination, height of the plant, biomass, and chlorophyll contents under salt stress that ameliorate the negative effects of soil salinity. Water potential and stomatal opening is a crucial plant physiological activity for their survival which even salinity-stressed condition were found to be modified by PGPR to compensate salt stress [121,122]. For example, *Enterobacter cloacae, Pseudomonas fluorescence, Bacillus pumilus, and Exiguobacterium aurantiacum* were found to greatly alleviate the toxic effect of salt stress in *Triticum aestivum* plants [122,123]. Moreover, Ali et al. [124] reported that under salt stress circumstances, *Enterobacter cloacae* PM23 boosted maize growth, biomass, photosynthetic pigment contents, carotenoids, and relative water content compared to control treatment. Similar effects were observed when co-inoculation of *Rhizobium* sp. and *Enterococcus mundtii* in *Vigna radiata* were carried out and obtained the grain production was improved under saline stress by regulating ion homeostasis [120]. Additionally, when infected with *B. megaterium*, *Solanum lycopersicum* and *Arabidopsis thaliana* both grew roots, shoots, and more leaves under salt stress [125,126]. Furthermore, *S. marcescens* inoculation enhanced *Triticum aestivum* shoot length, fresh weight, and chlorophyll (Chl) content [127]. Under saline stress conditions, the *Enterobacter cloacae* in *Brassica napus* enhanced seedling development [128]. Inoculating *Triticum aestivum* with *Pseudomonas fluorescens* led to similar outcomes, as did inoculating *Oryza sativa* with *Alcaligenes faecalis*, *B. pumilus*, and *Ochrobactrum* sp. [129]. In addition, the application of some PGPR has been shown to improve nodule formation and fix nitrogen in plants under salt stress [130]. For example, *Rhizobium* sp. and *Bradyrhizobium japonicum*’s co-inoculation improved root nodule formation in *Glycine max* compared to control conditions, resulting in increased stress tolerance, plant growth, and higher yield [130]. Likewise, *Bacillus aryabhattai* and *Azotobacter vinelandii* inoculation enhanced root nodule numbers and N-contents in *Trifolium repens* compared with the non-inoculated plants [131]. However, PGPR not only increased nodule numbers but also increased plant dry weight, shoot dry weight, the extent of nitrogen yield and protein content in some applications [132].

Many studies described that PGPR can alleviate the salt-induced growth inhibition of plants by positively regulating ion homeostasis and antioxidant enzyme activity, improving photosynthetic attributes, secondary metabolite accumulation, and oxidative stress reduction (Figure 1 and Table 4, [6,118,133,134]). For instance, the use of PGPR reduced the negative effects of salinity in pea (*Pisum sativum*) by enhancing the plants’ proline and soluble sugar contents while lowering sodium (Na^+^) contents, which in turn reduced the amount of electrolyte leakage and H_2_O_2_ content [135,136]. In addition, the harmful effects of salinity are reduced by PGPR via declining lipid peroxidation and ROS in wheat plants [137]. Singh et al. [125] and Kumawat et al. [120] reported that PGPR alters the selectivity of Na^+^, K^+^, and Ca^2+^ under salt stress and thus maintains ionic balance due to ion homeostasis. Moreover, inoculating *Pseudomonas* sp. or *Glutamicibacter* sp. with the halophte *Suaeda fruticosa* led to noticeably greater shoot dry weight and decreased buildup of Na^+^ and Cl^−^ in shoots of salt-treated plants [138]. Similarly, the *Piriformospora indica* inoculation in *Zea mays* decreased K^+^ flow from roots while increasing K^+^ concentration in shoots under saline condition; this effect may be linked to a high-affinity K^+^ transporter where PGPR produced a proton-driven force through H^+^-ATPase [139]. Moreover, *Azotobacter* isolates under salinity stress had greater K^+^/Na^+^ proportions in shoots and decreased Na^+^ and Cl^−^ amounts in maize leaves [140]. Additionally, some PGPR lowered Cl_2_ and NO_2_ concentrations and increased the K^+^/Na^+^ ratio, which contributed to enhanced stomatal conductance, and maintained hormonal balance and photosynthesis under salt stress [6,141]. It has also been reported that PGPR incorporation enhances the synthesis of the phytohormone that improves salt stress tolerance [6]. Such as, Some PGPR i.e., *Thalassobacillus denorans*, *Oceanobacillus kapialis*, *Pseudomonas* strains, *Bacillus tequilensis* and *Bacillus aryabhattai* synthesized more auxin and ABA, accumulated osmolytes in cell cytoplasm that sustain their cell turgor to make ensure plant growth under osmotic stress in *Oryza sativa* to endure high saline conditions [142]. In addition to the mechanisms mentioned above, in order to survive under salt stress, PGPR may alter salt tolerant gene expressions. The expression of TaABARE, TaOPR1, TaMYB, TaWRKY, TaST, SOS1, SOS4, TaNHX1, TaHAK, and TaHKT1 genes were up-regulated in PGPR inoculated plants leading to the expression of stress related genes [143,144]. According to the findings, salinity tolerance genes ZmNHX1, ZmNHX2, ZmN HX3, ZmWRKY58, and ZmDREB2A were up-regulated, as well as the antioxidants ZmGR1 (Zea mays glutathione reductase) and ZmAPX1’s (Zea mays ascorbate peroxidase) transcript levels [145,146]. Moreover, salinity tolerance was increased when PGPR enhanced antioxidant enzymes’ gene expression such as CAT, POD, APX, MnSOD, GR and GPX in inoculated plants [143]. Furthermore, according to Ali et al. [124], the inoculation of maize with *Enterobacter cloacae* PM23 increased APX, SOD, POD, total soluble sugars, and proteins while decreasing flavonoids and phenolic contents under salt stress. Additionally, in *Suaeda fruticosa* under high salinity, *Glutamicibacter* sp. inoculation dramatically decreased MDA levels while enhancing the activities of SOD, CAT, APX, and GR. Habib et al. [147] reported that salinity circumstances in the okra plant led to greater synthesis of APX and CAT by *B. megaterium* and *Enterobacter* sp. It has also been found that the treatment of *Arabidopsis* seedlings with *Enterobacter* sp. increased APX function and boosted salt tolerance [148]. Thus, from the reports of the studies it is apparent that the exogenous application PGPR could bring positive growth and yield results within the plants under saline condition and can be considered as a promising modern agronomic tactic to develop the plants survival under a saline environment. In future, dealing with extensive molecular research may reveal the efficacy of PGPR isolates and mechanisms to improve its stress responsive capability within the short duration in a wide area for sustainable agricultural production.

**Table 4:** Effects of PGPR on plant growth enhancement and salinity stress mitigation.

Plant Species	PGPR Inoculation	Salinity Stress	Effects of Inoculation	References
Wheat (*Triticum aestivum*)	*Pseudomonas fluorescence, Bacillus pumilus*, and *Exiguobacterium aurantiacum*	10% NaCl solution	Maximum root growth and dry biomass was observed; higher in proline and total soluble proteins contents; antioxidant activity improved; improved water and osmotic potential.	[122]
	*Enterobacter cloacae*	10% and 15% NaCl solution	Decreased the accumulation of Na^+^ and increased K^+^ uptake in shoots and roots; higher K^+/^Na^+^ ratios; improved antioxidant activity.	[123]
	*Bacillus subtilis* and *Arthrobacter* sp.	2–6 dSm^−1^	Improved antioxidant activity; increased in dry biomass, total soluble sugars and proline content.	[135]
	*Dietzia natronolimnaea*	100 and 150 mM NaCl	Modulated the expression of stress responsive genes; improved ion transporters TaNHX1, TaHAK, and TaHKT1; improved the activities of antioxidant enzymes.	[143]
	*Serratia marcescens*	150–200 mM NaCl	Higher osmo-protectants and growth parameters; higher K^+^/Na^+^ ratios; increased SOD, APX, and CAT activity.	[127]
Maize (*Zea mays*)	*Kocuria rhizophila*	100 and 200 mM NaCl	Improved IAA and the ABA activity; upregulation of salt tolerant genes ZmNHX1, ZmNHX2, ZmNHX3, ZmWRKY58 and ZmDREB2A; higher K^+^/Na^+^ ratios; improved the growth parameters; higher chlorophyll, proline, and total soluble sugar content.	[146]
	*Azotobacter chroococcum*	0, 2.93 and 5.85 g NaCl/kg soil	Increased in biomass and stomatal conductance; higher K^+/^Na^+^ ratios; improved antioxidant enzyme activity.	[140]
	*Enterobacter cloacae*	0, 300, 600, and 900 mM NaCl	Enhanced plant growth, biomass, and photosynthetic pigments under salinity stress; enhanced radical scavenging capacity, RWC, soluble sugars, proteins, secondary metabolite content.	[124]
	*Piriformospora indica*	500 μM KCl and 100 μM CaCl_2_	Higher biomass and stomatal conductance; lower K^+^ efflux from roots and higher potassium content in shoots.	[139]
Soybean (*Glycine max*)	*Rhizobium* sp. *Bradyrhizobium japonicum* and *Hydrogenophaga* sp.	100, 250, and 500 mM NaCl solution	Higher shoot biomass at the vegetative stage, reproductive stages; improved seed weight and shoot K^+^/Na^+^ ratio.	[130]
	*Methylobacterium aminovorans* and *Methylobacterium rhodinum; Bradyrhizobium japonicum* and *Bacillus megaterium*	0.170 dSm^−1^	Increased nodule numbers and dry weight of nodules; significantly increased in N, P and K; higher number of pods, seed index and seed yield.	[132]
Rice (*Oryza sativa*)	*Pseudomonas pseudoalcaligenes and Bacillus pumilus*	5, 10, 15, 20, and 25 g NaCl L^−1^	Reduced lipid peroxidation and superoxide dismutase activity; reduced plant cell membrane index cell caspase-like protease activity, and programmed cell death.	[137]
	*Bacillus amyloliquefaciens*	120 and 250 mM NaCl	Higher synthesis of amino acids; improved endogenous SA and ABA; improved plant physiology.	[142]
Mung bean (*Vigna radiate*)	*Rhizobium* sp. and *Enterococcus mundtii*	10% NaCl solution	Higher seed germination and seedling growth and biomass; enhanced chlorophyll content and macro-micronutrient uptake; improved soil physical, chemical and biological parameters.	[120]
Barley (*Hordeum vulgare*)	Bacillus megatherium, Pseudomonas fluorescens, Bacillus circulans, Paenibacillus polymyxa, Azotobacter chroococcum, *Azospirillum* sp. Paenibacillus polymyxa2, Azospirillum brasilense, *Hyderella* sp.	250, 500 or 1000 mM NaCl	Alleviated the deleterious effect of salinity; higher dry masses and relative water content.	[133]
Pea (*Pisum sativum*)	*Acinetobacter bereziniae, Enterobacter ludwigii,* and *Alcaligenes faecalis*	75 mM, 100 mM and 150 mM NaCl	Improved the growth parameters; higher chlorophyll, proline, and total soluble sugar content; improved electrolyte leakage; improved the activities of antioxidant enzymes.	[136]
Pepper (*Capsicum annuum*)	*Azospirillum brasilense* and *Pantoea dispersa*	40, 80 and 120 mM NaCl	Higher K^+^ /Na^+^ ratio; improved leaf photosynthesis and stomatal conductance.	[141]
Burclover (*Medicago* sp.)	*Bacillus megaterium, E. medicae, Ensifer* *Medicae* and *B. megaterium*	0–2000 mM NaCl	Improved IAA and the ACC deaminase activity; higher chlorophyll, proline, and total soluble sugar content.	[125]
Okra (*Abelmoschus esculentus*)	*Bacillus* *megaterium* and *Enterobacter* sp.	75 mM NaCl	Enhanced ROS-scavenging enzyme activity; increased antioxidant enzyme SOD, APX, and CAT; upregulation of ROS pathway genes CAT, APX, GR, and DHAR.	[147]
*Suaeda fruticosa*	*Glutamicibacter* sp. and *Pseudomonas* sp.	600 mM NaCl	Increased shoot K^+^ and Ca^2+^ content; lowered shoot MDA concentration and less accumulation of Na^+^ and Cl^−^ in shoots.	[138]
Rapeseed (*Brassica napus*)	*Enterobacter cloacae*	50 and 100 mM NaCl	Promoted seed germination and seedling growth; improved chlorophyll, water potential and other physiological activity.	[128]
*Avena sativa, Medicago sativa, and Cucumis sativus*	*Advenella incenata, Providencia re-* *Ttgeri, Acinetobacter calcoaceticus*, and *Serratia plymuthica*	Salinity stress	Enhanced ROS-scavenging enzyme activity; increased SOD, APX, and CAT activity; enhanced plant growth, and photosynthetic pigments. enhanced RWC and proteins, content	[145]
Tomato (*Solanum lycopersicum*)	*Bacillus megaterium*	200 mM NaCl	Improved the growth parameters and biomass; higher chlorophyll, proline, and total soluble sugar content.	[126]

## 3. Limitation of Organic Amendments and Future Perspectives

The organic amendments have particular physiochemical features and their application in soil has a great influence on soil properties as well as plant growth and development. It has been apparent from the above discussion that external organic activities operate as a powerful growth regulator, enhancing plant growth performance in salt-stressed environments. Although organic farming approaches are particularly beneficial for agricultural production in salt, they do have certain drawbacks.

- Its preparations, which are organically altered as natural weathering processes, need more labor, time, space, and raw resources.
- More experienced and skilled people, as well as scientific understanding, are required to maintain environmental conditions such as temperature, moisture, and respiration.
- Some organic methods, such as vermicomposting, biochar, and bio-fertilizer, emit a foul stench and attract flies. On the worm-feeding materials, harmful molds and bacteria are frequently produced in some cases.

Regardless of the fact that organic amendments take longer to prepare and require additional forms of management. In order to increase plant nutrition in salt-stressed agroecosystems, numerous proactive and preventive strategies have been used over time, with well-defined adverse effects. Numerous methods, such as organic amendments, have shown to be highly efficient in easing different agricultural restrictions, such as salt stress. Multidisciplinary approaches and solutions, driven not only by plant and agri-environmental scientists but also by experts from other fields (remote sensing, artificial intelligence, machine learning, big data analyses, etc.), can produce very helpful tools for detecting, guarding against, and controlling salinization, thereby minimizing the harm brought on by salt stress. However, many reports have been published about the role of organic amendments in the alterations of physio-biochemical reactions to plants under salt stress. To counteract salt stress, however, additional approaches and strategies based on breeding assisted by genetic markers, genome editing, and advanced biotechnological procedures can be applied in addition to all standard treatments. In addition, there is currently a paucity of knowledge regarding how secondary metabolites, distinct stress-responsive genes, and the primary metabolic pathways that govern due to salinity. More studies are required to better understand the morphological, physio-biochemical, transcriptomics, and proteomics of organic amendment application in a saline environment to improve crop productivity.

## 4. Conclusions

Abiotic stressors are significant barriers that reduce agricultural yields around the world. One of the most damaging environmental variables limiting agricultural productivity is salinity. Salinity-induced oxidative stress and Na+ ion absorption lead to cellular damage including ionic instability, which inhibits growth and has detrimental effects on the morphological and biochemical characteristics of plants. It is absolutely necessary to look for environmentally sound and long-term solutions to reduce the negative effects of salt on plants. However, these negative impacts of salinity were lessened by the applications of VC, VW, BC, BF, and PGPR. It is clear from the discussion that VC, VW, BC, BF, and PGPR promote plant growth and increase salt tolerance by maintaining ionic homeostasis, enhancing antioxidant enzyme activities, lowering osmotic and oxidative stress, and regulating gene expression, all of which lead to improved plant growth and productivity. Although several studies on the regulatory functions of VC, VW, BC, BF, and PGPR in various crops under salt stress have been carried out, there is still much that needs to be investigated at the molecular, biochemical, and physiological level.

## Data Availability

Not applicable.
